# Ribbon α-Conotoxin KTM Exhibits Potent Inhibition of Nicotinic Acetylcholine Receptors

**DOI:** 10.3390/md17120669

**Published:** 2019-11-28

**Authors:** Leanna A. Marquart, Matthew W. Turner, Lisa R. Warner, Matthew D. King, James R. Groome, Owen M. McDougal

**Affiliations:** 1 Department of Chemistry and Biochemistry, Boise State University, Boise, ID 83725, USA; leannabrown@u.boisestate.edu (L.A.M.); lisawarner@boisestate.edu (L.R.W.); matthewking990@boisestate.edu (M.D.K.); 2 Biomolecular Sciences PhD Program, Boise State University, Boise, ID 83725, USA; matthewturner1@u.boisestate.edu; 3 Department of Biological Sciences, Idaho State University, Pocatello, ID 83209, USA; groojame@isu.edu

**Keywords:** α-conotoxin, nicotinic acetylcholine receptor, NMR, two-electrode voltage clamp electrophysiology, PC12 cell, DockoMatic

## Abstract

KTM is a 16 amino acid peptide with the sequence WCCSYPGCYWSSSKWC. Here, we present the nuclear magnetic resonance (NMR) structure and bioactivity of this rationally designed α-conotoxin (α-CTx) that demonstrates potent inhibition of rat α3β2-nicotinic acetylcholine receptors (rα3β2-nAChRs). Two bioassays were used to test the efficacy of KTM. First, a qualitative PC12 cell-based assay confirmed that KTM acts as a nAChR antagonist. Second, bioactivity evaluation by two-electrode voltage clamp electrophysiology was used to measure the inhibition of rα3β2-nAChRs by KTM (IC_50_ = 0.19 ± 0.02 nM), and inhibition of the same nAChR isoform by α-CTx MII (IC_50_ = 0.35 ± 0.8 nM). The three-dimensional structure of KTM was determined by NMR spectroscopy, and the final set of 20 structures derived from 32 distance restraints, four dihedral angle constraints, and two disulfide bond constraints overlapped with a mean global backbone root-mean-square deviation (RMSD) of 1.7 ± 0.5 Å. The structure of KTM did not adopt the disulfide fold of α-CTx MII for which it was designed, but instead adopted a flexible ribbon backbone and disulfide connectivity of C2–C16 and C3–C8 with an estimated 12.5% α-helical content. In contrast, α-CTx MII, which has a native fold of C2–C8 and C3–C16, has an estimated 38.1% α-helical secondary structure. KTM is the first reported instance of a Framework I (CC-C-C) α-CTx with ribbon connectivity to display sub-nanomolar inhibitory potency of rα3β2-nAChR subtypes.

## 1. Introduction

Conotoxins are 10–50 amino acid peptide toxins present in the venom of predatory marine snails of the genus *Conus*. With sequence hypervariability and disulfide bond constrained scaffolds, conotoxins have a complex classification system of 28 superfamilies, segregated in part by activity on ion channels present in excitable tissues such as nerve and muscle [1]. α-Conotoxins (α-CTxs) target ligand-gated nicotinic acetylcholine receptor (nAChR) ion channels and inhibit ion flow by causing a dynamic structural change upon binding that results in the closing of the channel. nAChRs are pentameric transmembrane channel proteins that form different combinations of homo and heteropentameric subtypes, leading to a range of functions and ligand specificity across the host. nAChRs are found in many tissues including muscle and the central and peripheral nervous systems, and perform numerous physiological functions, such as the modulation of neurotransmitter release in the central nervous system by post and presynaptic excitation [2]. In the modulation of neurotransmitter release, acetylcholine activation of presynaptic nAChRs causes sodium influx and subsequent cellular depolarization, resulting in the activation of voltage-gated calcium channels and an influx of calcium ions that initiates a signaling cascade ending in the release of dopamine-containing vesicles. Because of their involvement in neurotransmitter release, nAChRs play a central role in the pathology of Parkinson’s disease and other neurological disorders. For example, Parkinson’s disease patients experience a degradation of neurons expressing α6α4β2β3-nAChRs and consequently display symptoms reflecting loss of function dependent on dopamine release. Targeting specific nAChR isoforms is a promising strategy in the development of improved drug therapies for Parkinson’s disease and other neurological diseases, including Alzheimer’s, Tourette’s, and Schizophrenia [3,4,5,6,7]. However, there still exists a gap in understanding of the mechanism of nAChR–ligand binding.

α-CTxs possess rigid scaffolds and are among the most potent inhibitors of nAChRs, making them insightful molecular probes for the elucidation nAChR binding paradigms. α-CTx structure–activity relationships have been developed for α-CTxs and their analogs, leading to the discovery of disease-relevant nAChR subtypes and the identification of new ligand–nAChR binding sites [8,9,10]. Model systems used to study nAChR–ligand binding include pheochromocytoma (PC12) cell assay [11], electrophysiology [12], mouse brain studies [13,14,15], and computational molecular dynamics simulations [9,16,17,18,19,20,21,22,23,24,25,26,27]. Expression systems for studying nAChRs in vitro often prove costly and complex [28,29,30,31], highlighting the potential value of computational studies. Emerging computational strategies can produce promising results for nAChR ligands, but potential small molecule drugs require evaluation by functional experimentation. Here, we present the validation results of a computational study that used the genetic algorithm managed peptide mutant screening (GAMPMS) program in DockoMatic v. 2.1 to predict the sequence for an α-CTx MII analog for optimal binding to the rat (r)α3β2 nAChR isoform; the outcome of the computational study was the designed peptide KTM [16,17,18].

**Figure 1:**
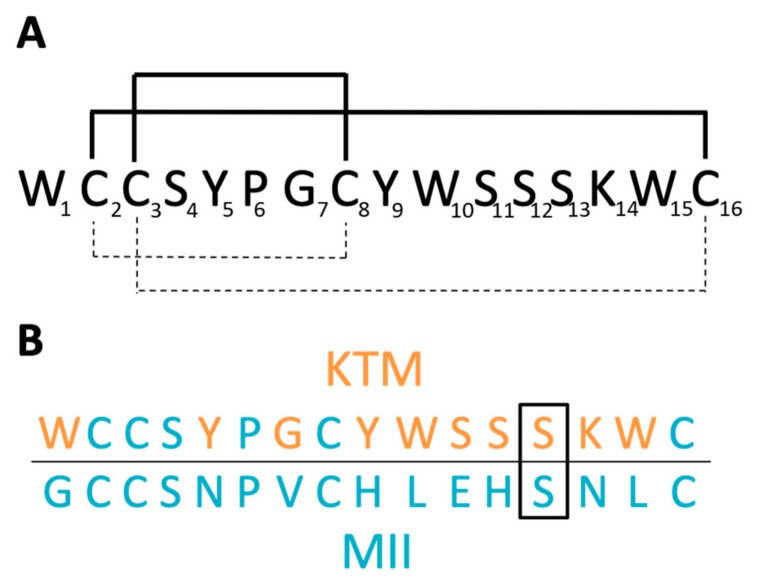
(**A**) Sequence of KTM with globular disulfide bond connectivity of C2–C8 and C3–C16 (dashed line), and disulfide bonding from oxidative folding following solid phase peptide synthesis of ribbon connectivity C2–C16 and C3–C8 (solid line). (**B**) Sequence comparison of KTM to α-CTx MII. Of the 10 residues in α-CTx MII that were allowed to vary in the genetic algorithm managed peptide mutant screening (GAMPMS) algorithm (orange), all but one amino acid (S13, boxed) changed to give the sequence of KTM.

DockoMatic is an open source program with an intuitive user interface to run software applications for ligand and receptor structure file creation, perform high throughput virtual screening, and output docking results ranked in order from best to worst binding affinity [19,20,21,32,33,34]. DockoMatic uses the highly innovative GAMPMS algorithm specifically designed for peptide library creation, and can correlate peptide structure to drug identity by way of the small-molecule peptide-influenced drug repurposing (SPIDR) utility that permits screening of molecular databases using a template structure derived from a peptide scaffold [16,17,18]. Previously, the GAMPMS algorithm was used to evaluate the binding affinity of over 41 billion combinations of α-CTx MII mutants for a homology model of the rα3β2 nAChR isoform [17]. α-CTx MII was selected as a template for this study because it has been well characterized as a very potent and selective inhibitor of the rα3β2 nAChR isoform. α-CTx MII is a 16 amino acid peptide with the primary sequence GCCSNPVCHLEHSNLC and disulfide bonds between C2–C8 and C3–C16. The globular structure of α-CTx MII is characterized by an α-helix initiated at P6 and ending at H12, providing the peptide with approximately 40% α-helical content. The GAMPMS program was used to change the primary sequence of α-CTx MII to create a peptide library of nearly 41 billion unique α-CTxs [17]. All amino acids were varied in the primary sequence with the exception of the four cysteine residues required to form disulfide bonds, and the conserved proline at position 6 required for initiation of the α-helix. Molecular docking in DockoMatic ranked the peptides by highest binding affinity for the rα3β2 nAChR isoform. The top fifty mutant peptides with highest affinity for the receptor were compared for amino acid identity at each site in the peptide primary sequence to generate a consensus peptide. Figure 1A,B shows the sequence and disulfide connectivity of KTM (A), and the sequence comparison of KTM to α-CTx MII (B). The consensus peptide, given the arbitrary name KTM (Figure 1A; dashed), represents a rationally designed peptide ligand that was computationally predicted to have enhanced binding affinity for the rα3β2 nAChR isoform. 

The sequences of α-CTx MII and KTM both contain 16 amino acids (Figure 1B). α-CTx MII has a Framework I cysteine pattern of CC-C-C, consisting of two disulfide bonds between C2–C8 and C3–C16, making it a globular connectivity pattern, and a 4/7 loop sequence common to many α-CTxs [35]. The design of KTM was inspired by the pharmacological features present in α-CTx MII and was anticipated to adopt the same globular disulfide connectivity. KTM activity on nAChRs was assessed qualitatively by an in vitro cell-based PC12 luminescence assay, and quantitatively for inhibition of rα3β2 nAChR isoforms using two-electrode voltage clamp electrophysiology. KTM demonstrates remarkable sub-nanomolar inhibition of selective nAChR subtypes, commensurate with that of α-CTx MII. In the present study, we show that synthetic KTM produced by undirected folding preferentially forms ribbon C2–C16 and C3–C8 disulfide linkages. In their review, Akondi, et al. suggest that correct folding of synthetic peptides is critical to the maintenance of biological activity [35], but in the case of KTM, the globular disulfide pattern is not the same as that found in α-CTx MII, but rather resembles that of the ribbon isomer of α-CTx AuIB [36,37]. The net effect of the disulfide connectivity is that α-CTx MII adopts a globular scaffold, while KTM is consistent with ribbon-connectivity. Solution nuclear magnetic resonance (NMR) spectroscopy was used to determine the three dimensional structure of the synthesized KTM for comparison to α-CTx MII and the computationally predicted structure. Molecular dynamics simulations in Gromacs were used to assess peptide dynamics.

## 2. Results

### 2.1. Bioactivity

**Figure 2:**
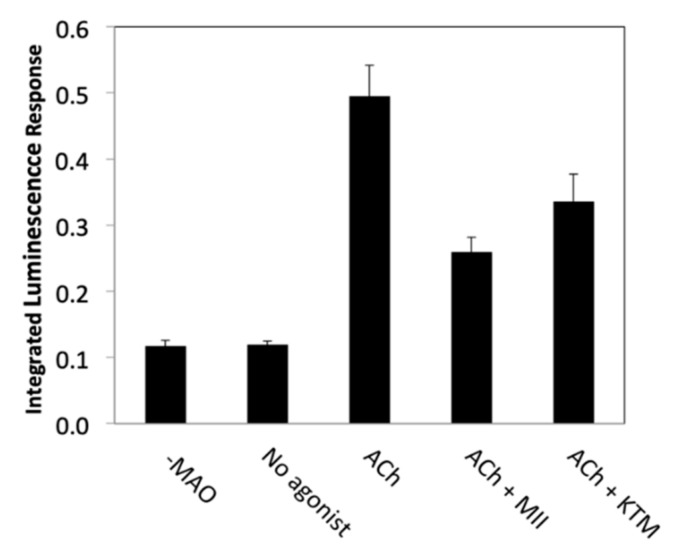
Qualitative PC12 assay luminescence responses upon stimulation of nicotinic acetylcholine receptors (nAChRs) with acetylcholine (ACh), with and without 10 µM toxin. Assays performed with the addition of α-CTx MII and KTM resulted in a diminished luminescence recording compared to an ACh control.

KTM was qualitatively evaluated for nAChR bioactivity using a PC12 cell assay; antagonist activity by KTM in the presence of acetylcholine (ACh) was confirmed (Figure 2), prompting quantitative evaluation of bioactivity by electrophysiology. In the preliminary screening qualitative assay, ACh is used to stimulate PC12 cells, opening nAChR channels, resulting in dopamine release [38,39]. Following release, dopamine is oxidized by monoamine oxidase, generating hydrogen peroxide that catalyzes a chemiluminescence reaction involving luminol and horseradish peroxidase, producing a detectable response. Incubation with α-CTxs prior to stimulation with ACh inhibits nAChRs, resulting in a diminished signal, as is observed for treatment with α-CTx MII (see Figure 2). A decreased signal resulting from treatment with KTM indicates that nAChRs are inhibited by this compound.

**Figure 3:**
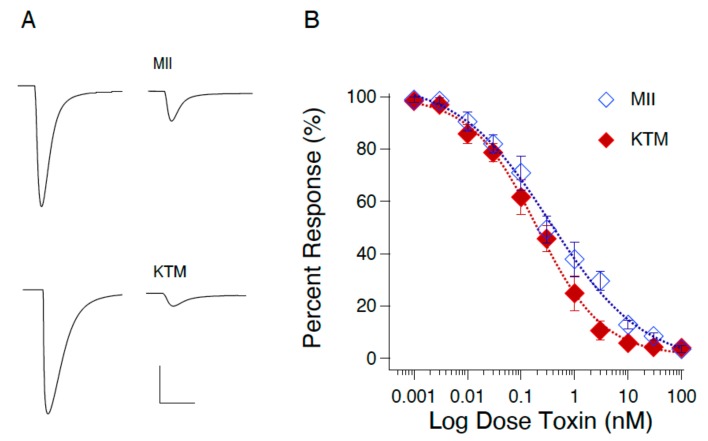
Responses to the local application of ACh for 30 ms are shown for control and after 3 nM toxin application; calibration horizontal 2 sec, vertical 1 µA (**A**). Concentration-dependent response curves for blocking rα3β2 nAChR by α-CTx MII (red) and KTM (blue) (**B**). Hill coefficients for the concentration response curves of α-CTx MII and KTM are 0.5 and 0.7, respectively. IC_50_ values of KTM and α-CTx MII are 0.19 ± 0.02 nM and 0.35 ± 0.08 nM, respectively. Data are means ± SEM from eight to 12 trials.

Two-electrode voltage clamp experiments were used to determine the IC_50_ for KTM on rα3β2 nAChRs expressed in *Xenopus laevis* oocytes. Figure 3A shows responses to local application of ACh prior to (left) and following (right) toxin exposure. The concentration-dependent curves for inhibition of rα3β2 nAChR by MII and KTM are shown in Figure 3B. KTM exhibited potent inhibition with an IC_50_ of 0.19 ± 0.02 nM commensurate with α-CTx MII with an IC_50_ of 0.35 ± 0.08 nM. α-CTx MII and KTM have Hill coefficients of 0.5 and 0.7, respectively.

### 2.2. Structure Determination

**Figure 4:**
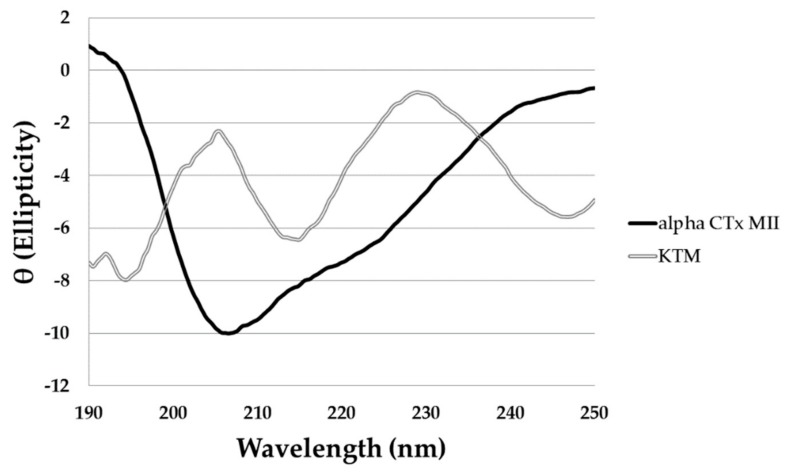
The circular dichroism (CD) spectrum of α-CTx MII (solid black line) and KTM (double lined grey). Measurements for each peptide were taken in water at 50 µM, and a pathlength of 1 mm. The α-helical content of α-CTx MII and KTM were estimated to be 38.1% and 12.5%, respectively, as calculated from the observed signal at 222 nm.

Analysis of the circular dichroism (CD) spectrum for KTM (Figure 4, double lined grey) gave a predicted α-helical content of 12.5%, consistent with the ribbon-type isomer fold (C1–C4; C2–C3), and not the expected globular-type fold (C1–C3; C2–C4) characteristic of α-CTx MII, for which the α-helical content is 38.1% (Figure 4, solid black line). The large negative peak commonly observed for α-CTxs corresponds to the α-helical portion of the peptide, and is predominantly absent in the CD spectrum of KTM. The interpretation of CD spectra for flexible small peptides is representative of an ensemble of conformations, so it is difficult to draw definitive secondary structure conclusions based solely on CD data. The CD data in Figure 4 did identify variation in the secondary structure between KTM and α-CTx MII that brought into question the disulfide connectivity in KTM, necessitating framework determination for KTM by partial reduction mass spectrometry.

Partial reduction by TCEP of 100 pmol of synthetic KTM peptide gave expected product profiles in LC-MS chromatograms with mass increases corresponding to partial reduction (+2 m/z) and alkylation (NEM, +125 m/z; IAA, +59 m/z) (see Materials and Methods). Sequence analysis showed the disulfide bridging pattern was not consistent with the expected α-CTx C2–C8/C3–C16 globular linkage as found in α-CTx MII, but rather a C2–C16/C3–C8 ribbon linkage (Figure S1), as observed in α-CTx AuIB [36,37].

**Figure 5:**
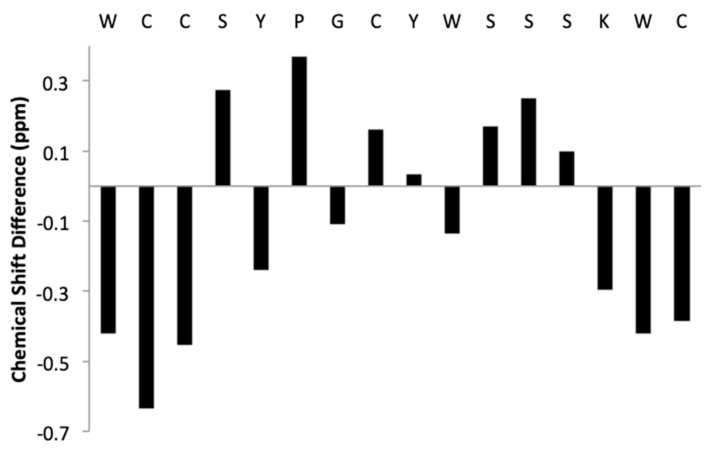
Chemical shift difference between α-protons of amino acids in KTM and predicted random coil chemical shifts for the same amino acids.

**Figure 6:**
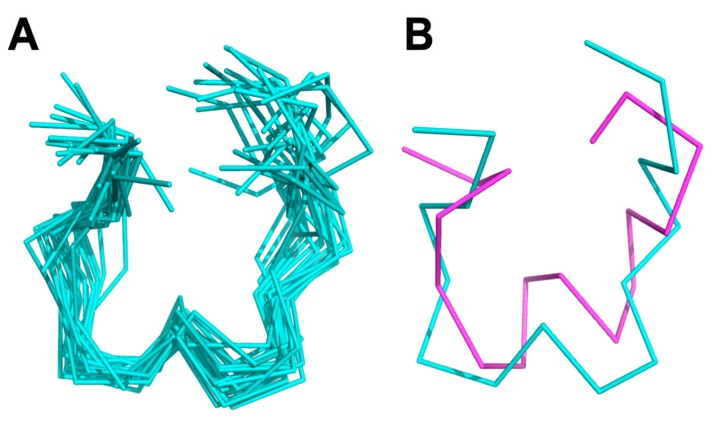
NMR solution structure of KTM, where (**A**) is a ribbon representation of an ensemble of the 20 lowest energy structures, with an average RMSD to the mean structure of 1.7 ± 0.4 Å, and (**B**) represents an overlay of the mean calculated structure from NMR (cyan) and the computationally predicted structure (magenta) that have an average RMSD of 3.5 Å.

NMR structure determination for KTM was performed to compare the computationally predicted C2–C8/C3–C16 globular structure to the synthesized C2–C16/C3–C8 ribbon structure. Assignment of ^1^H resonances for KTM was achieved using standard methods [40]. A combination of COSY, TOCSY, and NOESY spectra in both 30% ACN/70% water and 30% ACN/70% D_2_O were used to reduce ambiguities in assignment. Fifteen amino acid spin systems were assigned in the fingerprint region (7.6–8.8 ppm), and the final amino acid, P6, was identified in the α-proton region (5.2–3.8 ppm). Table 1 shows the chemical shift assignments for each of the sixteen amino acids in KTM, and Figure 5 shows the calculated random coil chemical shift difference. Table 2 shows the 32 nuclear Overhauser effect (NOE) distance restraints, four dihedral angles, and two disulfide bond constraints that were input into CYANA for structure calculation. NMR structure determination using CYANA [41] confirms the peptide backbone exists as a ribbon, lacking a defined α-helix. Despite a reasonably rigid scaffold constrained by two disulfide bonds, the backbone structure of KTM reflects the influence of side chain mobility particularly evident for the aromatic Tyr and Trp residues. Figure 6 A-B shows a comparison of the NMR-derived structures (A), and an overlay of the median NMR-derived structure (cyan) to the computationally predicted structure (magenta) for KTM (B). The root-mean-squared deviation (RMSD) for backbone atoms to a mean structure was calculated to be 1.7 ± 0.4 Å (Figure 6A). The RMSD between the NMR solution structure of KTM and the computationally predicted KTM structure was 3.5 Å, which is expected considering the computationally predicted structure maintained the globular disulfide connectivity consistent with α-CTx MII (Figure 6B).

### 2.3. Molecular Dynamics Simulations

**Figure 7:**
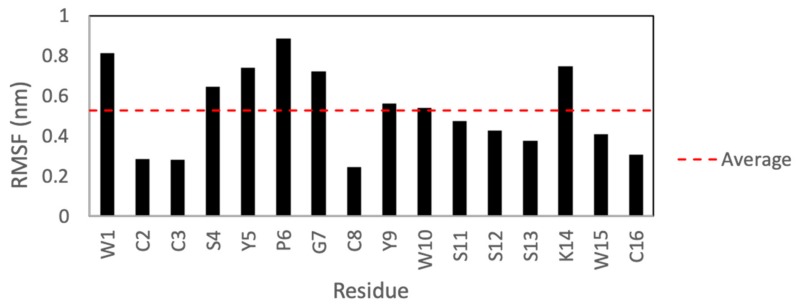
Root mean square fluctuations for each of the 16 residues of KTM over a 50 ns MD simulation. Residues W1, S4–G7, and K14 were observed to exhibit the greatest root-mean-square fluctuations (RMSFs).

Figure 7 shows the root-mean-square fluctuations (RMSFs) for each residue after a 50 ns molecular dynamics simulation in Gromacs. According to these results, the side chains of KTM are expected to have a high degree of fluctuation. Residues W1, S4–G7, and K14, particularly, show the highest degrees of fluctuation.

## 3. Discussion

Qualitative evaluation of KTM bioactivity with the PC12 assay confirmed that KTM reduced the amount of dopamine released from the cells, presumably by blocking nAChRs (Figure 2) [11]. To assess whether KTM acted on rα3β2-nAChRs, as it was designed to do, quantitative evaluation of its bioactivity was performed by two-electrode voltage clamp electrophysiology using *Xenopus* oocytes expressing rα3β2-nAChRs. KTM caused blockage of rα3β2-nAChRs with an IC_50_ of 0.19 ± 0.02 nM as compared to 0.35 ± 0.08 nM for α-CTx MII (Figure 3). The similar efficacy of KTM compared to that of α-CTx MII supports the premise that the synthetic peptide is an effective antagonist of rα3β2-nAChR.

KTM exhibited potent inhibition of rα3β2-nAChRs commensurate with α-CTx MII, necessitating validation of synthetic peptide structure for comparison to the computationally predicted structure. The primary sequence of KTM differs from α-CTx MII with regard to nine of the sixteen amino acids. First, the NMR structure of KTM was determined using the method established by Wüthrich [40]. Sequence, distance, dihedral angle, and disulfide bond restraints were entered into CYANA for structure calculation. It was anticipated that the disulfide bonds in synthesized KTM, that were formed by undirected folding, and those of native α-CTx MII, would be consistent. Despite extensive analysis of NMR data and restraint assignment, CYANA failed to provide an ensemble of KTM backbone structures that converged better than a RMSD of 2.5 Å. An evaluation of the CD spectrum and partial reduction mass spectrometry data provided definitive evidence that the disulfide pattern for KTM synthesized by undirected folding was not the same as the globular fold of α-CTx MII (C1–C3, C2–C4), but rather was consistent with the ribbon fold observed for an isomer of α-CTx AuIB (C1–C4, C2–C3) (see Figure S1). An ordered secondary structure commensurate with the common α-CTx 4/7 loop peptide α-helix of MII was not expected due to the α-helical content for KTM interpreted as 12.5% based on CD spectrum, which is much lower than the α-helical content for α-CTx MII of 38.1% (Figure 4). Upon changing the disulfide bond connectivity restraints in CYANA, a final set of 20 KTM structures with an RMSD among backbone atoms of 1.7 ± 0.5 Å was obtained with no distance restraint or conformation violations detected (Figure 6). The RMSD between the NMR solution structure of KTM and the computationally predicted structure of KTM was 3.5 Å. The rough similarity in backbone shape between the computationally predicted and NMR solution structure generated for KTM, despite differing disulfide linkages, is suspected to be the reason for the observed nAChR bioactivity. Preliminary analysis of NMR data for chemical shift deviation from random coil was used as an indication of expected KTM peptide rigidity (Figure 5). Random coil peptides generally show amide proton chemical shifts between 8.09 and 8.45 ppm [42], while those in KTM were between 7.64 and 8.74, indicating regions of structure rigidity. Similarly, random coil peptides generally show α-proton chemical shifts between 4.4 and 4.8 ppm, while those in KTM were between 4.07 and 5.33 ppm. Thus, the chemical shift variation observed for amide and α-protons in KTM (Figure 5) were consistent with data expected for a peptide of reasonably rigid scaffold.

**Figure 8:**
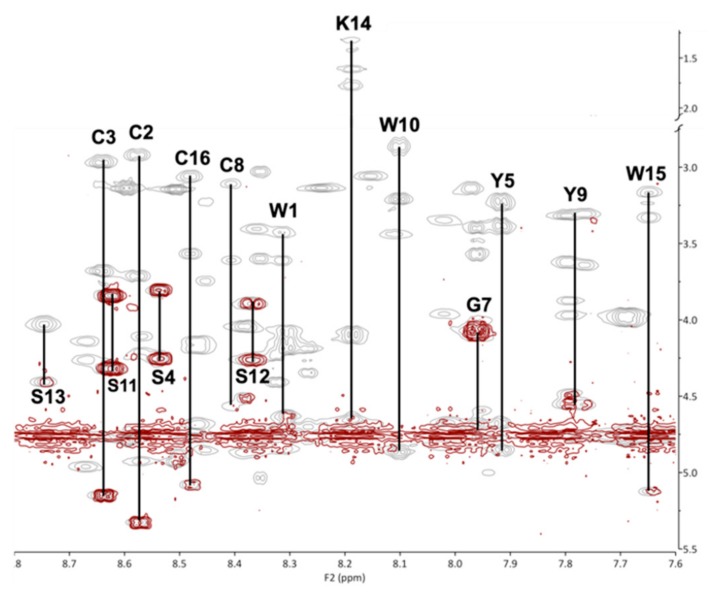
Fingerprint region of COSY (red) and TOCSY (grey) spectra overlaid for residues 1–5 and 7–16, acquired at 600 MHz for KTM at 298 K in 30% ACN/70% H_2_O. Residue assignments are indicated by their one-letter amino acid code.

The relatively low number of distance and angle constraints for KTM was due to challenges associated with a 1942 Da peptide and the high redundancy of amino acids (4C, 4S, 3W, 2Y), resulting in many proton chemical shifts in similar or identical electronic environments. The interpretation of NMR spectra for KTM was severely complicated due to peak overlap, ring-flipping of aromatic side chains, and spectral shift between H_2_O and D_2_O spectra. Figure 8 shows an overlay of the fingerprint region for COSY and TOCSY spectra used to assign the spin systems in KTM from which the chemical shift assignments as summarized in Table 1 were generated. The proton chemical shifts for aromatic amino acids are largely missing from Table 1 due to assignment ambiguity originating from the three tryptophan and two tyrosine residues with overlapping chemical shifts further exacerbated by ring flipping. Additional structure challenges arose from the molecular weight of KTM correlating to the NOE detection minimum, limiting the number of medium- and long-range NOEs that could be assigned as restraints. Conotoxin structure determination by NMR is inherently difficult due to weak NOE signals, limited distance for protons to provide an assignable NOE, and side chain mobility that provides conformations where protons enter in and out of detectable proximity to one another [43]. Additionally, the high number of heavy aromatic side chains in KTM is suspected to contribute to inherent flexibility.

**Figure 9:**
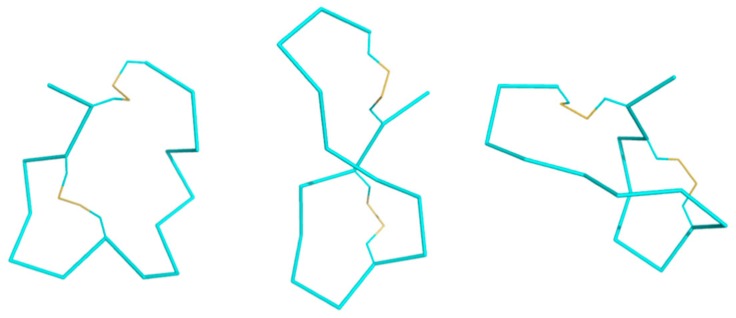
Ribbon structures of KTM at 0 ns (left), 25 ns (middle), and 50 ns (right) in the molecular dynamics simulation. Loop 2 appears to swing across the peptide over the course of the simulation.

Perhaps the most striking finding of this study was that KTM, which was predicted to act on rα3β2-nAChRs, did indeed do so, and with very high potency, despite having a ribbon-type α-CTx disulfide connectivity. There is precedent for ribbon isomers of Framework I α-CTxs with binding affinity for rα3β2-nAChRs. Dutton, et al. (2002) characterized a non-native ribbon disulfide bond isomer of recombinant α-CTx AuIB that, while more flexible than the native globular isomer, exhibited 10 times more potent activity with nAChRs in rat parasympathetic neurons than the native globular isomer [36,37]. They presumed that the flexibility of the ribbon isomer allowed the peptide to adopt a complementary conformation with the receptor binding site. KTM is the first example of a Framework I α-CTx with ribbon fold to demonstrate sub-nanomolar inhibition of rα3β2-nAChRs. To briefly explore why KTM exhibits functionality consistent with α-CTx MII, a comparison of the electrostatic maps of α-CTx MII and KTM was performed using the ABPS Electrostatics plugin in PyMOL [44] after performing a 50 ns molecular dynamics simulation in Gromacs. The simulation shows that the side chains for W1, S4–G7, and K14 have a high degree of fluctuation (Figure 7), and that loop 2 is especially dynamic (Figure 9), indicating that a single model may not well represent the peptide. 

**Figure 10:**
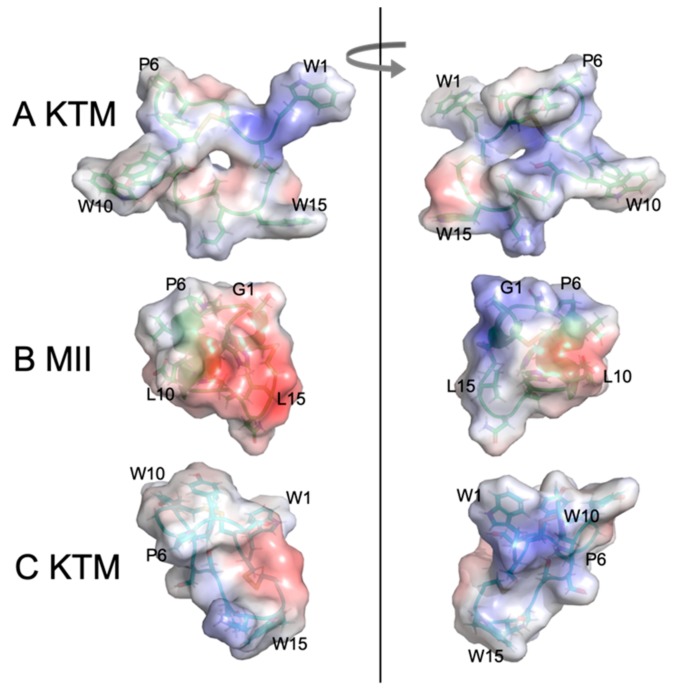
Electrostatic maps of KTM at 0 ns of the molecular dynamics simulation (**A**), α-CTx MII (**B**), and KTM at 50 ns of the molecular dynamics simulation (**C**). Images on the left and right are rotated by 180 degrees.

Figure 9 shows the progression from left to right of representative structures through the course of a molecular dynamics simulation with snapshots taken at 0, 25, and 50 ns run time. The dynamic movement of the second loop is exemplified throughout the course of the MD trial. To illustrate the dynamic flexibility of KTM and its potential for induced fit into the binding site of rα3β2 nAChR, the electrostatic surfaces of KTM at 0 ns (Figure 10A) and at 50 ns (Figure 10C) are shown in comparison to α-CTx MII (Figure 10B). KTM can adopt both a structure with a surface volume much larger than α-CTx MII with multiple points of protrusion from aromatic side chains and a hollow core (Figure 10A), as well as a more compact structure that more closely resembles that of α-CTx MII as loop 2 appears to flex inward (Figure 10C). 

Figure 10 shows the convergence from NMR-derived structure (0 ns MD, A) to computationally refined KTM conformation upon 50 ns simulation (C), compared to the structure of α-CTx MII (B). The MD simulation provides correlation between KTM and MII that reflects common surface electrostatics and structure topography attributes that may be used to explain the observed potent activity of KTM on nAChRs (Figure 3). 

## 4. Materials and Methods 

### 4.1. Synthesis

KTM was synthesized by and purchased from CS Bio (Menlo Park, CA) in folded form as a white powder. Following solid phase peptide synthesis, disulfide linkages were allowed to form into the most thermodynamically stable conformation under reducing conditions. The major isomer was collected by HPLC at ca. 98.36% purity. The peptide was collected at 10.71 min using a 20%–50% gradient of Buffer B (0.1% TFA in ACN) over a 20 min run time, where Buffer A was 0.1% TFA in H_2_O. The column was a Phenomenex Luna C_18_ with specifications of 5 µM, 100 Å, 4.6 ×250 mm. The flow rate was 1 mL/min and the injection volume was 20 µL. Peptide identity was confirmed by mass spectrometry where the expected molecular weight was 1942.21 Da and the found molecular weight was 1941.52 Da.

### 4.2. Disulfide Bond Analysis

Analysis was performed using an UltiMate 3000 HPLC (Thermo Scientific, Waltham, MA, USA) equipped with a Corona Veo RS charged aerosol detector (CAD), an UltiMate 3000 Diode Array Detector (DAD), and MSQ Plus mass spectrometer (MS). HPLC separation of peptide species was achieved using an Acclaim 120 C_18_ column (2.1 × 150 mm, 3 µm) (Thermo Fisher), and mobile phases consisting of 0.1% formic acid (v/v) in water (Buffer A) and 0.1% formic acid (v/v) in acetonitrile (Buffer B) with a flow rate of 0.3 mL/min. A linear gradient method beginning at 85% Buffer A and 15% Buffer B, up to 60% Buffer B over a 25 min run time achieved desired separation from a 10 µL sample injection volume. Data were analyzed with the Chromeleon 7.2 Chromatography Data System (Thermo Fisher). The partial reduction strategy of Gray [45] was used to sequentially reduce and alkylate disulfide bonds. Synthetic KTM peptide (0.1 nmol) from CS Bio was dissolved in 1 M equivalent of tris 2-carboxyethyl phosphine (TCEP) in 25 mM ammonium formate, at pH 4.5, and incubated at 37 °C for 10 min. Following incubation, the reaction mixture was separated by HPLC, and the peptide reduction was monitored by mass spectrometry through observation of a mass increase of +2 m/z, corresponding to the peptide with one disulfide bond reduced. Two molar equivalents of N-ethylmaleimide (NEM) were added and the reaction mixture and alkylation was monitored by LC-MS for observation of a mass increase of +125 m/z. The increase in m/z of +125 in the doubly charged ion equates to a mass shift of +250 Da in KTM corresponding to the coupling of one thiol to NEM. Peptide fractions, as identified by UV absorbance at 280 nm, were collected manually into 1.5 mL polypropylene centrifuge tubes and lyophilized to dryness. The resultant di- and tetra-alkylated peptides appeared as dull white powders. Monocyclic intermediates were reconstituted in 1:1 (v/v) acetonitrile/25 mM ammonium formate, reduced with dithiothreitol (DTT) at a final concentration of 10 mM for 1 hr at 37 °C, and alkylated with iodoacetamide (IAA) at a final concentration of 55 mM for 1 hr in the dark at room temperature. The reaction mixture was injected onto the LC-MS to monitor reduction and alkylation by observation of a mass increase of +59 m/z, corresponding to the addition of acetamide. Doubly alkylated peptides were submitted for sequence analysis to determine the locations of S-carboxamidomethyl-L-cysteine residues, and confirm the disulfide connectivity (see Figure S1). MS/MS fragmentation for sequence analysis was achieved using a Velos Pro Dual-Pressure Linear Ion Trap mass spectrometer (Thermo Scientific) coupled to a Nanoscale LC system at a flow rate of 300 nL/min. A fused silica emitter directly attached to the analytical column through a zero dead volume union was used to spray peptides at a voltage of 2.2 kV. MS/MS data was collected in data-dependent acquisition mode, using collision-induced dissociation (CID) with a normalized collision energy of 35% to fragment the precursor ions. MS/MS data for the 10 most abundant precursor ions was selected from the proceeding full MS scan over the m/z range of 300–2000. Proteome Discoverer 2.2 (Thermo Scientific) was used to analyze the data.

### 4.3. Circular Dichroism Spectropolarimetry

Circular dichroism (CD) spectra were recorded on a Jasco J-810 spectropolarimeter (Jasco, Inc., Easton, MD, USA) with a cell path length of 0.1 cm at room temperature in a nitrogen atmosphere in water. Scans were acquired from 190 to 250 nm. The bandwidth of 1 nm, a speed of 50 nm/min, and a resolution of 0.5 nm were used. A total of 5 scans per sample were averaged, baselines were subtracted, and the sample was run in triplicate. A final concentration of 50 µM of each sample was used. Analysis and data processing were carried out with the Jasco system software and Microsoft Excel. Mean residue ellipticity (*θ*MRE, in deg × cm^2^ × dmol^−1^) for each spectrum was calculated from the formula *θ*MRE = *θ*/(10C_r_ × *l*), where *θ* is the measured ellipticity in millidegrees, C_r_ is the molar concentration, and *l* is the path length in centimeters. The α-helical content was estimated from the formula *θ*MRE = 30300fH–2340, where fH is the fraction of α-helical content (fH × 100, expressed as a percentage) calculated from the *θ*MRE at 222 nm, which is a widely used proxy for helical secondary structure that is useful to assess disruption of the secondary structure, as was performed here [46,47,48].

### 4.4. Nuclear Magnetic Resonance Spectroscopy

NMR samples were prepared at a concentration of approximately 3 mM in either 30% deuterated acetonitrile (d-ACN)/70% water or 30% d-ACN/70% deuterium oxide (D_2_O). d-ACN was used due to the limited solubility of KTM in water. D_2_O samples were prepared by dissolving lyophilized sample in 30% d-ACN/70% D_2_O solvent and immediately acquiring spectra. Two-dimensional ^1^H NMR experiments and spectral interpretation were performed by established methods [40,41,42,49,50,51,52,53,54].

All NMR data were acquired on a 600 MHz Bruker Avance III NMR spectrometer at 298 K. COSY, TOCSY, and NOESY spectra were acquired using water suppression for aqueous samples. A series of NOESY spectra were acquired with mixing times of 150, 200, 250, 300, and 350 ms. TOCSY spectra were acquired with an 80 ms mixing time (30% ACN/60% H_2_O /10% D_2_O). Parameter set details are provided in Table S1. Spectra were processed in Topspin and analyzed in CCPNMR v2 and v3 [55] to correct resonance shifts, manually pick peaks, and identify spin systems.

#### 4.4.1. Restraint Set Generation

Three-bond ^1^H^N^-^1^H^α^ coupling constant values were determined by visual inspection of a high-resolution 1D ^1^H spectrum in Topspin. Backbone dihedral phi angle restraints were set to −120 ± 40 for ^3^*J*_HH_^1^H^N^-^1^H^α^ coupling constant values greater than 7.5 Hz and to −65 ± 25 for ^3^*J*_HH_^1^H^N^-^1^H^α^ coupling constant values less than 5 Hz [54]. Inter- and intraproton distance ranges were calibrated within CYANA. Distance restraints were derived from NOESY spectra recorded at 298 K and mixing time of 350 ms. 

#### 4.4.2. Structure Calculation

Input files were formatted for the CYANA program (v 2.1). The autoassign script in CYANA was used for assignment of 69 hydrogen atom chemical shift values. A chemical shift tolerance of 0.075 was used for both axes. Disulfide bonds were used as distance restraints. CYANA produced an ensemble of 20 top structures based on 32 restraints. The final set of 20 structures gave a root-mean-square deviation among backbone atoms of 1.7 ± 0.4 Å. The structure most representative of the mean structure was determined with WHATIF [56]. Procheck [57] and Verify3D [58] online servers were used to assess structure quality. Molprobity scores are reported in Table S2 [59].

### 4.5. PC12 Assay

Cell culture and bioactivity assessment were performed according to Marquart, et al. [11]. The PC12 assay was performed using a Biotek Synergy H1 microplate reader (Winooski, VT). All chemicals were purchased at the highest purity available (>95%) from Fisher Scientific. ATCC^®^ CRL1721™ PC12 cells were provided by the Biomolecular Research Center at Boise State University.

#### 4.5.1. Cell Culture

PC12 cells were cultured into laminin-coated flasks and triturated to detach. Cells were grown in a T-75 flask until dense enough to plate onto a laminin-coated flat-bottom well plate and treated with starvation media (30 ng/mL NGF 2.5 S, 1 µM nicotine) for 3–4 days before performing the assay. 

#### 4.5.2. Assay 

Cells were washed with Locke’s solution before introducing the assay solution, which included 10 µM toxin, 0.8 ng/mL POD, 25 ng/mL MAO, and 50 µM luminol. The luminol was added to quench any dissolved oxygen in the cell environment and the assay solution. Cells were allowed to equilibrate for 5–10 min before the addition of 2 mM luminol and 50 µM ACh, for a total well volume of 100 µL. Upon equilibration, the luminescence signal was detected. (-)MAO and (-)cells were used as negative controls. Assays were performed at 37 °C, detecting luminescence for 1–2 min per well. Luminescence response curves were then integrated as a measure of dopamine secretion inhibition resultant from peptide treatment for analysis.

### 4.6. Electrophysiology

#### 4.6.1. rα3β2-nAChR expression in *Xenopus laevis* oocytes

cDNA preparation, oocyte harvest, expression of nAChR subunits, and culture were performed as described previously [60]. The rat isoforms of neuronal nAChR subunits α3 and β2 in vectors pSP64 and pSP65, respectively, were generously provided by Dr. Steven Heinemann (Salk Institute, San Diego, CA). All salts and antibiotics were obtained from Sigma or Fisher Scientific. Oocytes were extracted from adult *Xenopus* according to protocols approved by the Institutional Animal Use and Care Committee at ISU and according to AAALAC guidelines. Oocytes were isolated with 2 mg/L collagenase and cultured at 17.5 °C in sterile ND-96 saline containing 96 mM NaCl, 2 mM KCl, 1.8 mM CaCl_2_ 1 mM MgCl_2_, and 5 mM 4-(2-hydroxyethyl)-1-piperazineethanesulfonic acid (HEPES), with 5 mM Na pyruvate, 2% horse serum, 100 mg/L gentamicin, 100 mg/L amikacin, 50 mg/L ciprofloxacin, 20 mg/L tetracycline, and 100 U/L streptomycin/penicillin, at pH 7.4. Messenger RNA was transcribed as detailed previously; α3 and β2 mRNA was coinjected at 41 nL into oocytes 3 to 5 days prior to recordings.

#### 4.6.2. Two-Electrode Voltage Clamp

To record the response to ACh, oocytes were impaled with glass electrodes containing 3 M KCl for two-electrode voltage clamp electrophysiology. Oocytes were placed in a 300 µL recording chamber and perfused at 1.0 mL/min with ND-96 saline containing atropine sulfate (1 mM), at room temperature. Voltage clamp was performed with an OC-725C amplifier (Warner Instruments, Hamden, CT) with data acquisition achieved through an ITC-18 interface and PatchMaster 2.35 software (HEKA Instruments, Bellmore, NY). Oocytes were held at -70 mV between trials, and at -80 mV during trials of ACh application. During perfusion, a 20 to 50 ms pulse of ACh chloride (0.01 M, Sigma, St. Louis, MO) in the bath solution was locally applied from a glass capillary tube positioned above the oocyte, with pressure ejection using a PicoSpritzer II valve controller (General Valve Corporation, Fairfield, NJ). Once a reliable response was obtained, the chamber was perfused with α-CTx MII or KTM for 15 min, and the ACh pulse was applied. Inward sodium current amplitudes for each toxin trial were normalized against the control response to ACh as percent response. Each data point of the dose response curve represents the average value of 8 to 12 measurements. Dose-dependent response curves were fit to Equation (1):

%response = 100/[1 + ([toxin] / IC_50_)*^n^*], (1)

where *n* is the Hill coefficient and IC_50_ is the inhibitory concentration at half-maximal block, by non-linear regression analysis using Igor Pro 6.37 (Wavemetrics, Lake Oswego, OR, USA).

### 4.7. Molecular Dynamics Simulations

Molecular dynamics simulations were performed with the AMBER03 force-field parameter set [61] using the Gromacs 2019 software package [62]. The median conformation for KTM from the top 20 NMR solution structures was selected as the input structure for MD simulation in Gromacs. Since the NMR spectra were acquired for a sample in 30% acetonitrile/70% water, the solvated box was obtained by filling a dodecahedral box with the appropriate number of acetonitrile molecules to achieve the desired mixed solvent proportions. A topology for acetonitrile was created for this purpose. The shortest distance of peptide atoms from the box boundary was 1.2 Å. Simulations were carried out at 300 K in a periodic box with a minimum-image convention. The integration step was 1 fs, and total duration of each run was 50 ns. Water, acetonitrile, and ions were temperature coupled. Energy minimizations and N, V, T and N, P, T equilibrations were performed before running the simulation. A 500 ps equilibration was conducted under the NVT ensemble using a velocity-rescale thermostat at 300 K with a coupling time constant of 0.1 ps. A subsequent 500 ps NPT equilibration was performed using the isotropic Berendsen barostat with a time constant of 0.1 ps. Simulations and models were visualized in PyMOL [44].

## 5. Conclusions

This investigation sought to validate the accuracy and usefulness to rationally design a selective antagonist of a specific nAChR isoform based on the well characterized marine drug α-CTx MII. Synthetic KTM peptide was confirmed to be a potent sub-nanomolar inhibitor of rα3β2 nAChRs. It was expected that KTM would fold into the same C1–C3 and C2–C4 disulfide pattern, consistent with α-CTx MII. Instead, the folding of KTM resulted in a C1–C4 and C2–C3 ribbon Framework I α-CTx, consistent with the recombinant isomer of α-CTx AuIB [36,37]. It has been stated in the literature that correct folding of synthetic peptides is critical to the maintenance of biological activity, but in the instance of KTM, the globular disulfide pattern is not the same as that found in α-CTx MII, but rather resembles the ribbon-connectivity studied for α-CTx AuIB [36,37]. KTM is the first example of sub-nanomolar nAChR antagonism by a Framework I α-CTx of ribbon-connectivity. A compelling next step for this work would be the directed folding of KTM to assess whether the potency of the globular-connectivity Framework I peptide may be further enhanced for nAChR inhibition. Validation of computationally predicted peptide binding and biological activity may lead to a better understanding of molecular probes for the treatment of neurological diseases like Parkinson’s Disease. Drugs that selectively target human α6α4β2β3 nAChR isoforms could be used as new therapies with fewer side effects than existing treatments. The current study offers a workflow to begin the search for drug therapies inspired by marine natural products.

## Figures and Tables

**Table 1:** Proton chemical shift assignments for amino acids in KTM.

Residue	NH	αH	βH	Other
W1	8.31	4.62	3.61,3.44	2H 7.37, N1H 10.23
C2	8.57	5.33	3.71,2.92	
C3	8.63	5.14	2.96,3.67	
S4	8.53	4.23	3.97	
Y5	7.91	4.84	3.39,3.23	
P6		4.07	1.99,2.20	δH 3.70,3.58
G7	7.96	4.08		
C8	8.40	4.53	3.62,3.11	
Y9	7.78	4.57	3.32,3.60	
W10	8.09	4.84	3.21	2H 7.74, N1H 10.52, 7H 7.88
S11	8.62	4.33	3.83	
S12	8.38	4.25	3.88	
S13	8.74	4.40	4.01	
K14	8.19	4.66	1.72,1.56	δH 1.39, γH 1.29, εH 2.91, N2H 8.00
W15	7.64	5.12	3.13,3.31	2H 7.48, N1H 10.33
C16	8.47	5.08	3.57,3.04	

**Table 2:** Nuclear magnetic resonance (NMR) restraints used in CYANA, and the resulting structure statistics for KTM.

Experimental Data	
**Distance Restraints**	
Total NOE	32
Intra-residue	12
Inter-residue	20
Sequential	18
Short range	30
Medium range	2
Long range	0
ϕ Dihedral angle restraints	4
Disulfide restraints	2
Total NOE violations exceeding 0.3 Å	0
Total NOE violations exceeding 0.3 Å	0
**Structure Statistics**	
Average pairwise RMSD (Å)	
Backbone atoms (residues 1–16)	1.7 ± 0.5
Heavy atoms (residues 1–16)	3.0 ± 0.7
Ramachandran statistics	
%Favored and allowed regions	100
%Disallowed regions	0

## Supplementary Materials

The following are available online at https://www.mdpi.com/1660-3397/17/12/669/s1, Table S1: Parameter set details for NMR experiments.; Table S2: Molprobity scores for NMR ensemble; Figure S1: Annotated MS-MS spectrum of relevant distinguishing B and Y fragments.
