# Supplementary material for: Ribbon α-Conotoxin KTM Exhibits Potent Inhibition of Nicotinic Acetylcholine Receptors
Source: Mar Drugs. 2019 Nov 28;17(12):669. doi: 10.3390/md17120669 (PMC6950571; doi:10.3390/md17120669)
Supplement: Supplementary file 1 [file marinedrugs-17-00669-s001.pdf]

Supplemental Materials

NMR Assignments

Table S1 provides parameter set details for all NMR experiments performed.
Assignment of spin systems to the amino acid primary sequence was accomplished as follows: Spin systems were identified by confirming the presence of H-βH NOESY peaks and βH-βH NOESY peaks. All spin systems were confirmed on both sides of the diagonal.

The fingerprint region (**Figure 8A**) revealed an obvious lysine spin system pattern unique to any other residue in the peptide, clearly identifying K14. This assignment was used as a handle for other assignments. The W15 and C16 spin systems were then easily identified by two large NOESY peaks with the K14 NH resonance in the NH region. The W15 spin system was distinguished between the C16 system by a clear NOESY peak between the NH resonance and an N2H resonance in the downfield N2H region. S13 was identified based on a clear NOESY between the S13 NH resonance and the K14 NH resonance in the NH region. S12 was then identified by a clear NOESY between the S13 and S12 NH resonances in the NH region.

The α proton of P6 at 4.07 ppm was easily identified in the upfield α-β region by its spin system pattern unique to any other residue in the peptide. This assignment was also used as a handle for other assignments. G7 was also clearly identified by its unique spin system. The C8 spin system was confirmed by an NH-NH NOESY peak to G7, then Y9, W10, and S11 were identified in the same manner. The remaining unassigned serine spin system was then assigned as S4 by elimination. These assignments were fortified by corresponding aH-NH/βH-NH NOESY peaks between adjacent residues. C2 and C3 were predicted to have similar environments. As expected, the remaining two appropriate spin systems were identified at nearly overlapping chemical shifts, showing clear NH-NH and NH-aH NOESY peaks. The only remaining unassigned spin system for the peptide was then identified as W1 by elimination. C2 was distinguished from C3 by an NH-NH NOESY peak to W1. Additional inter-residue NOESY peaks were then identified based on these assignments.

Table S1. Parameter set details for NMR experiments.

Sample	Spectrum	pulse program	mix (ms)	Spectral width F1 (Hz)	Spectral width F2 (Hz)	scans	size of fid (F1)	size of fid (F2)
30% ACN/ 70% H_2O	noesy	noesyfgpphwg	50	8417.5	8417.5	64	256	2048
	noesy	noesyfgpphwg	150	8417.5	8417.5	64	256	2048
	noesy	noesyfgpphwg	250	8417.5	8417.5	64	256	2048
	noesy	noesyfgpphwg	350	8417.5	8417.5	64	256	2048
	tocsy	mlevgpphw5	80	6002.4	7194.24	64	300	6144
	cosy	cosygpprqf	n/a	7215	7211.5	32	600	8192
	hsqc	hsqcetgpsi	n/a	25000	7812.5	256	200	1024
30% ACN/ 70% D_2O	noesy	noesygpphpr	50	8417.5	8417.5	64	256	2048
	noesy	noesyfgpphwg	150	8417.5	8417.5	64	256	2048
	noesy	noesyfgpphwg	250	8417.5	8417.5	64	256	2048
	noesy	noesyfgpphwg	350	8417.5	8417.5	64	256	2048
	tocsy	mlevphpr	80	6002.4	7194.24	64	300	6144
	cosy	cosygpqf	n/a	7211.5	7211.5	32	600	8192
	hsqc	hsqcetgpsi	n/a	25000	7812.5	256	200	1024

Molprobity Scores

Molprobity scores for all 20 models in the NMR ensemble are reported in **Table S2**. Clash scores and Ramachandran outliers were zero for all models, with no bad bonds or angles and no Cβ deviations >0.25Å.

Table S2. Molprobity scores for the 20 NMR ensemble structures. The lowest percentile was 76th for model 10.

Model	Molprobity Score	Percentile
1	1.13	99
2	1.77	86
3	1.15	99
4	1.33	98
5	0.50	100
6	2.06	73
7	1.33	98
8	1.77	86
9	1.15	99
10	2.00	76
11	0.50	100
12	1.15	99
13	1.33	98
14	1.15	99
15	0.50	100
16	1.77	86
17	1.15	99
18	1.95	78
19	1.33	98
20	1.77	86

Partial Reduction MS-MS

B and Y fragments were analyzed as the most accurate fragments for MS-MS fragmentation. Relevant fragments identified that distinguished a C2-C16/C3-C8 disulfide connectivity over a C2-C8/C3-C16 connectivity are shown in **Figure S1**.

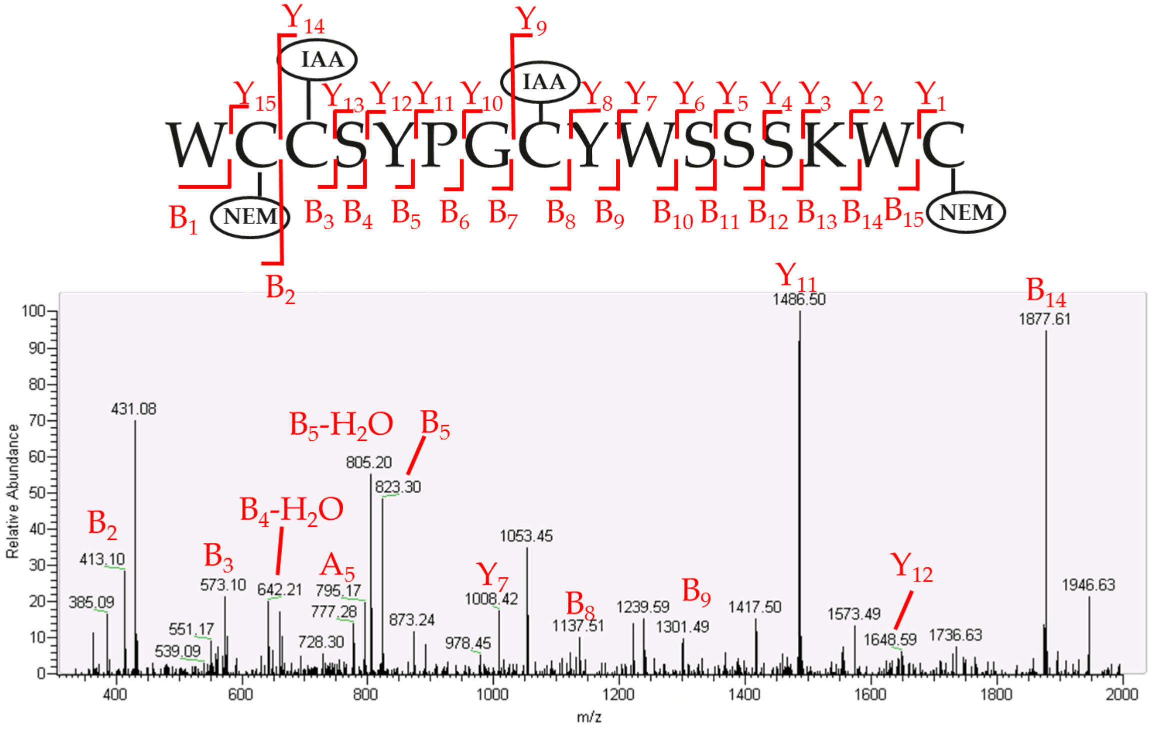

Figure S1. Annotated MS-MS spectrum of relevant distinguishing B and Y fragments. Fragments are numbered along the KTM sequence, showing position of corresponding alkylations with N-ethylmaleimide (NEM) and iodoacetamide (IAA).
